# Development of an Immunochromatographic Strip Using Conjugated Gold Nanoparticles for the Rapid Detection of *Klebsiella pneumoniae* Causing Neonatal Sepsis

**DOI:** 10.3390/pharmaceutics13081141

**Published:** 2021-07-26

**Authors:** Noha M. Elhosseiny, Tamer M. Samir, Aliaa A. Ali, Amani A. El-Kholy, Ahmed S. Attia

**Affiliations:** 1 Department of Microbiology and Immunology, Faculty of Pharmacy, Cairo University, Cairo 11562, Egypt; ahmed.attia@pharma.cu.edu.eg; 2 Department of Microbiology and Immunology, College of Pharmaceutical Sciences and Drug Manufacturing, Misr University for Science and Technology, Cairo 12573, Egypt; tamer.mohamed@must.edu.eg; 3 Department of Pediatrics, Faculty of Medicine, Cairo University, Cairo 11562, Egypt; draliaaadel.aa@kasralainy.edu.eg; 4 Department of Clinical Pathology, Faculty of Medicine, Cairo University, Cairo 11562, Egypt; aelkholy@kasralainy.edu.eg; 5 School of Pharmacy, Newgiza University, Giza 12588, Egypt

**Keywords:** *K. pneumoniae*, neonatal sepsis, immunochromatographic strip, diagnosis, rapid, gold nanoparticles

## Abstract

Neonatal sepsis is a leading cause of death among newborns and infants, especially in the developing world. The problem is compounded by the delays in pinpointing the causative agent of the infection. This is reflected in increasing mortality associated with these cases and the spread of multi-drug-resistant bacteria. In this work, we deployed bioinformatics and proteomics analyses to determine a promising target that could be used for the identification of a major neonatal sepsis causative agent, *Klebsiella pneumoniae*. A 19 amino acid peptide from a hypothetical outer membrane was found to be very specific to the species, well conserved among its strains, surface exposed, and expressed in conditions simulating infection. Antibodies against the selected peptide were conjugated to gold nanoparticles and incorporated into an immunochromatographic strip. The developed strip was able to detect as low as 10^5^ CFU/mL of *K. pneumoniae*. Regarding specificity, it showed negative results with both *Escherichia coli* and *Enterobacter cloacae*. More importantly, in a pilot study using neonatal sepsis cases blood specimens, the developed strip selectively gave positive results within 20 min with those infected with *K. pneumoniae* without prior sample processing. However, it gave negative results in cases infected with other bacterial species.

## 1. Introduction

The consensus definition for sepsis in neonates (1 week to 1 months of age) is a systemic inflammatory response (SIRS) associated with infection [1]. A number of clinical signs were set to define the occurrence of SIRS, including a core temperature of >38.5°C or < 36 °C, bradycardia, an elevated mean respiratory rate, and an abnormal leucocyte count [1]. This should be accompanied by a clinically proven infection, traditionally a positive culture as the “gold standard” for infection diagnosis [2]. “Severe” sepsis is a case associated with organ dysfunction [1].

Sepsis is one of the children’s leading cause of death worldwide [3], with a prevalence of 15.6% [4,5], and mortality rates of 9% to 20% [5,6]. It is estimated by the World Health Organization (WHO) that sepsis kills around 1 million newborns each year [7]. The burden is especially high for low and middle-income countries (LMICs), or the “developing” world, where severe neonatal sepsis could cause mortality rates of up to 40% [8]. Yet the problem remains with severe worldwide repercussions, even in the developed world. Four out of every 10 infants suffering from sepsis would eventually die [9], and in the United States, 36% of preterm neonates would suffer at least one episode of bloodstream infection while at the hospital, recording up to 50% mortality rate [10].

The most common causative agents for that type of sepsis include colonizers of the human birth canal, as well as those causing a maternal infection that could be transmitted transplacentally. This group includes most importantly *Streptococcus agalactiae* (GBS), *Candida* spp, *Listeria monocytogenes*, and non-typeable *Haemophilus influenzae* [11]. Microorganisms associated with hospital environment transmission include coagulase-negative staphylococci (CoNS), *Staphylococcus aureus*, *E. coli*, *Candida* spp, and *E. cloacae* [11,12]. These leading causes of neonatal sepsis vary depending on the region and time frame surveyed, as well as socioeconomic levels [13]. The *Klebsiella* species is one of the most common causative agents of neonatal sepsis, especially in LMICs [14,15,16]. The pathogen is especially problematic in the Neonatal Intensive Care Units (NICUs) of Egyptian hospitals accounting for up to 61.3% of neonatal sepsis cases in some settings [17,18,19,20]. Effective timely interventions are hampered by the rise of extensively drug resistant isolates, in addition to the difficulty in obtaining a definitive diagnosis of sepsis. Blood cultures are used as the definitive diagnosis for an active infection, but their reliability is hampered by the duration needed (24–48 h). Inoculation with the small blood volumes indicated for neonates (0.5–1 mL) also compromise the accuracy of the test and increase false negative rates [21]. The detection of sepsis-specific biomarkers is an attractive alternative to the time-consuming blood cultures. Some studied sepsis-biomarkers include complete blood picture, C-reactive protein, serum amyloid A, lipopolysaccharide-binding protein, Interleukin 6 (IL-6), Interleukin 8 (IL-8), Tumor Necrosis Factor alpha (TNFa), procalcitonin, and Cluster of Differentiation 163 (CD163) [22]. PCR-based detection has also been employed, achieving rapid detection, yet the cost is usually prohibitive especially in economically-challenged settings [23].

Immunochromatographic strips (ICS) as diagnostic tests are easy to use and produce, rapid, cost-effective, and could be used as point-of-care tests by healthcare personnel [24]. Recently they have been increasingly attractive as diagnostic tools for microbial infections, especially when rapid diagnosis could be life-saving, such as with neonatal sepsis, and SARS-CoV-2 infections [25,26]. In this study, we design and evaluate a prototype immunochromatographic strip for the diagnosis of neonatal sepsis, employing *K. pneumoniae*-specific surface-exposed peptide as a target for nanogold-conjugated antibodies.

## 2. Materials and Methods

### 2.1. Ethics Statement

Animal procedures were approved by the Research Ethics Committee of the Faculty of Pharmacy, Cairo University (MI-1612 & 2426) following the Guide for the Care and Use of Laboratory Animals published by the Institute of Laboratory Animal Research (Washington, DC, USA).

### 2.2. Bioinformatic Analyses of K. pneumoniae Proteome to Identify Potential Targets

The proteome of *K. pneumoniae* strain ATCC 13883 was retrieved from the NCBI (accession JOOW00000000), converted into fasta format, and submitted into the PSORTb server, for protein subcellular localization prediction (https://www.psort.org/psortb/, accessed on 15 August 2019) [27]. Proteins with a predicted “outer membrane” localization were further analyzed for species conservation and specificity. To determine if a protein was conserved across the species, its amino acid sequence was used as query for a blast analysis (https://blast.ncbi.nlm.nih.gov/Blast.cgi?PAGE=Proteins, accessed on 12 September 2019) [28] limited to *K. pneumoniae* as the search target. To determine if the protein is specific to *K. pneumoniae*, the same procedure was followed, except that the search was performed with the exclusion of *K. pneumoniae*. Results of the blast analysis were downloaded as multiple sequence alignments in Clustal format and examined using Jalview (https://www.jalview.org/, accessed on 7 October 2019) [29]. Regions of high and low conservation achieved with strains of the same species and other species, respectively, were determined, followed by a topology prediction using PRED-TMBB (http://bioinformatics.biol.uoa.gr/PRED-TMBB/input.jsp, accessed on 10 October 2019) [30] to determine the degree of surface exposure. Regions achieving all the criteria of conservation, specificity, and surface exposure were analyzed for immunogenicity using the Immune Epitope Database (IEDB) (http://www.iedb.org/, accessed on 27 October 2019) [31].

### 2.3. Validation of the Expression of the Selected Candidates via RT-RT-PCR

The cells of *K. pneumoniae* were grown logarithmically in Luria Bertani broth (LB, LabM, Lancashire, UK) at 37 °C with shaking at 180 rpm till reaching an OD_600_~0.4, and then the collected cells were incubated with either phosphate-buffered saline (PBS), pH 7.4 or normal human serum (NHS) (10% *v*/*v*) for two hours at 37 °C. RNA was then extracted using the RNeasy mini kit (Qiagen, Hilden, Germany), followed by reverse transcription with the QuantiTect reverse transcription kit (Qiagen). For RT-RT-PCR, the produced cDNA was used as a template for the reaction using the GoTaq^®^ qPCR SYBR Green master mix (Promega, Madison, Wisconsin, USA), and primers AA717 (Fw-5′-ACCTTGCTTGGTGGCATAA-3′) and AA718 (Rv-5′- GGTAAAGAGAGGTCGTTGGAAG-3ʹ) for amplification of the *K. pneumoniae* DR88_397 gene. The 16S rRNA was used as a normalizer with primers AA715 (Fw-5′-ACGGCCGCAAGGTTAAA-3′) and AA716 (Rv-5′-GTGGATGTCAAGACCAGGTAAG-3′). The specificity of the primers was confirmed using a melt curve analysis. To check for DNA contamination, no RT reactions were also tested.

### 2.4. Synthesis of the Target Peptide

The chosen peptide fragment from the *K. pneumoniae* protein encoded by gene DR88_397 designated KP_397(50-68) was synthesized by GenScript (Piscataway, NJ, USA). The purity of the peptide was assessed by HPLC using Alltima^TM^ C18 5 µm, 4.6 × 250 mm column, mobile phase A: 0.065% TFA in water & B: 0.05% TFA in acetonitrile, at flow rate of 1 mL/min and detection at 220 nm. The identity of the peptide was confirmed using mass spectroscopy. The sample was dissolved in 50% methanol (*v*/*v*), analysis was carried out by direct infusion using electrospray ionization in positive mode (Nebulizing Gas Flow: 1.5 L/min, Drying Gas Flow: 5 L/min, T. Flow: 0.2 mL/min, B. conc: 50%H_2_O/50%MeOH, CDL Temp: 250 °C, and Block Temp: 200 °C). The solubility of the peptide was also tested against a variety of solvents including ultrapure water and DMSO.

### 2.5. Immunization and Purification of Polyclonal Antibodies

Five female BALB/c mice, 20–22 gm in weight, (Theodor Bilharz Research Institute, Giza, Egypt) were immunized with two gradient doses of the synthesized peptide. The immunization regime followed was sensitizing dose of 100 µL 1:1 emulsion of 100-µg KP_397(50-68) peptide in Complete Freund’s Adjuvant (day 0), booster dose of 100 µL 1:1 emulsion of 50-µg peptide in Incomplete Freund’s Adjuvant (day 22). In parallel, mice were injected with emulsions of phosphate buffered saline (PBS) as the negative control. The initial dose and the booster were injected subcutaneously under anaesthesia with 2,2,2-tribromoethanol (200 µL of 25 mg/mL). The mice were sacrificed on day 37, using an overdose of 2,2,2-tribromoethanol (750 µL of the aforementioned dose), and blood was collected by exsanguination of the posterior vena cava. Immunoglobulins from the collected anti-sera were purified using the protein A agarose purification kit (KPL) according to the manufacturer’s instructions. The titers of the purified antibodies were assessed using an indirect ELISA assay, using the KP_397(50-68) peptide/*K. pneumoniae* cells (prepared as described before [32]) as a coat, and goat anti-mouse IgG, horseradish peroxidase (HRP)-labelled (Seracare, Milford, MA, USA), as the secondary antibody conjugate. Finally, the purified antibody fractions of the highest titers were pooled, concentrated, and the concentrations were determined using the BCA protein assay kit (Pierce, Waltham, MA, USA). Aliquots were stored at −20 °C for subsequent use.

### 2.6. Synthesis and Characterization of Gold Nanoparticles (GNPs)

The citrate reduction method was used for the synthesis of the GNPs. Briefly, sodium citrate solution (0.1 M) was added to boiling hydrogen tetrachloroaurate (III) (HAuCl_4_·3H_2_O) (0.1 mM) with vigorous agitation. This was followed by boiling for 10 min and further agitation for 15 min. The reaction yielded a wine-red solution that was cooled to room temperature and then stored at 4 °C in a light-resistant, 50 mL bottle. Spectrophotometric scanning of the produced particles in 2-fold serial dilution was performed using a Synergy 2 (BioTek, Winooski, Vermont, USA) multi-mode plate reader screening for the λ_max_ of absorption in the range between 400 and 800 nm. The mean particle size of the nanoparticles was determined by Photon Correlation Spectroscopy using a Malvern Zetasizer Nano-ZS (Malvern Instruments, Surrey, UK). Measurements of particle size were done in duplicate at a 90° angle, at 25 °C and the samples were diluted 1:10 using sterile water for injection. The zeta potential of the gold nanoparticle suspension was measured in mV by the Malvern Zetasizer Nano-ZS in duplicate to determine the surface charge and the potential physical stability of the system. Samples were diluted 1:10 in sterile water for injection at a 120° angle at 25 °C. The polydispersity index (PDI) was determined using the Malvern Zetasizer Nano-ZS. Size distribution analysis was carried out using electron microscopy and the prepared suspension was examined by a Jeol transmission electron microscope (TEM) 2000 fx. A total of two batches of GNPs were prepared; an initial preliminary small-scale one was followed by a main large-scale one. The data presented in this report for the GNPs characterization is representative of the main large-scale one with which most of the work for developing the ICS was performed.

### 2.7. Preparation of GNP-Antibody Conjugate

The protocol for preparing the GNP-antibody conjugate was adopted as previously described [33] with slight modifications. To determine the antibody-binding capacity of the prepared GNPs, a serial dilution of bovine serum albumin (BSA), ranging from 40 μg/mL to 10 mg/mL was performed. A 10 μL aliquot of each concentration was added to 50 μL of the prepared GNP solution, and the mixture was incubated for 30 min at room temp with gentle shaking. Following the incubation, 60 μL of a 20% solution of NaCl was added to each BSA-GNP mixture to challenge the conjugate stabilization, and the absorption was measure for each sample using a spectral scan analysis protocol in a Synergy 2 multimode plate reader (BioTek). The least concentration of BSA where no color change is observed after the NaCl addition contains enough protein for total coverage of the colloidal gold particles and hence for their stabilization.

To prepare the anti-KP_397(50-68) antibody-GNP conjugate, the pH of the GNP solution was adjusted to 8–9 using a solution of K_2_CO_3_ (0.1 M). The pH-adjusted GNP solution was slowly added to the antibody solution, with mild agitation, in a ratio of 5:1, respectively, so that the final concentration of the antibody in the mixture is 200 μg/mL. The pH of the mixture was measured again, and if necessary, adjusted back to a value of 8–9 with 0.1 M K_2_CO_3_. The mixture was then incubated for 30 min at room temperature, followed by the addition of 10% BSA in a 20 mM borax buffer (pH = 9.2) to achieve a final BSA concentration of 1%. The mixture was incubated at room temperature for 15 min, followed by centrifugation at 10,000× *g*, 4 °C for 30 min. Finally, the conjugate was resuspended in a 1% BSA, 2 mM borax buffer (pH = 9.2), equivalent to the initial starting volume of the GNP-antibody mixture and stored in a light-protected container at 4 °C until further use.

### 2.8. Assembly of the Immunochromatographic Strip

A glass fiber strip (5 mm × 15 mm) (Millipore, Burlington, MA, USA) was pre-soaked in the release agent (0.5% Tween-20, 0.5% bovine serum albumin, in water) for 5 min at room temperature, then dried in a 37 °C incubator for 2 h. The GNP-anti-KP_397(50-68) conjugate was mixed with the dispensing agent (20 mM PBS, 5% methanol, 0.1% lactose) in a ratio of 4:1 (*v*/*v*) then deposited on the dried glass fiber strip and left to dry in a 37 °C incubator for 2 h. A nitrocellulose membrane with an 0.45 μm pore size (Carl Roth) was cut into strips (5 mm × 30 mm) and the capture antibody (1.9 mg/mL), and control antibody (1 mg/mL Goat anti-mouse IgG, (KPL) mixed with 0.135 M sodium chloride (1:1 *v*/*v*), diluted 1/5 in dispensing solution to a final concentration of 100 μ g/mL before use) were dispensed in thin lines with a 10 μL micropipette tip on the membranes at a distance of approximately 10 mm and 15 mm, respectively. The membranes were allowed to dry at 37 °C for 10 min, then blocked by carefully immersing in the blocking solution (1% dried milk powder, 0.1% sodium azide) and incubated at room temperature for 30 min. The membranes were rinsed (3 × 10 s) in distilled water, and then allowed to dry at room temperature for 30 min. The cellulose fiber sample pad (5 mm × 15 mm) (Millipore), the treated glass fiber strip, the treated membrane strip, and the absorbent pad (5 mm × 10 mm) were all assembled as described before [33].

### 2.9. Determination of the Sensitivity and Specificity of the Constructed ICS

The constructed ICS was evaluated using a 2-fold serial dilution of the synthesized KP_397(50-68) peptide (156 μg/mL down to 9.76 μg/mL). A 160-μL aliquot of each concentration of the polypeptide was applied onto the sample pad and allowed to flow for 30 min before taking the result. Appearance of two bands at both the capture and control lines indicated a positive result, while the appearance of one band at the control line indicated a negative result. Sterile PBS was used as a negative control. The strip was also evaluated against whole bacterial cells. The bacterial cells suspensions were prepared as follows: An overnight culture of *K. pneumoniae* cells was used to inoculate LB broth in a 1:100 ratio. The cultures were incubated with shaking at 180 rpm, and 37 °C till an OD_600_ of 0.55–0.59 was reached. The cell pellets were washed twice with sterile PBS, and then resuspended in PBS so that the final inoculum adjusted corresponded to 10^8^ CFU/mL. The inoculum count was confirmed using viable count. For strip testing, a serial dilution of inoculum (10^8^ to 10^2^ CFU/mL) was prepared and dispensed in 200 µL aliquots. For specificity testing, the strip was challenged with a suspension of similarly prepared *E. coli* and *Enterobacter cloacae* cells (10^6^ CFU/mL).

### 2.10. Assessment of the Ability of the Developed ICS to Detect K. Pneumoniae in Neonatal Sepsis Blood Samples

Blood specimens collected from neonates diagnosed with neonatal sepsis due to *K. pneumoniae* infections confirmed by VITEK 2 were diluted 10-fold using sterile PBS (pH 7.4) and 200 µL of the diluted samples were loaded onto the sample pad of the assembled ICS. The sample was allowed to flow for 15–30 min before recording the test result. In addition, blood specimens from neonates diagnosed with neonatal sepsis due to *S. aureus* and CoNS were also tested to further assess the specificity of the developed ICS using the intended specimen type.

## 3. Results

### 3.1. In Silico Identification of Promising Protein/Peptide Candidates

**Figure 1:**
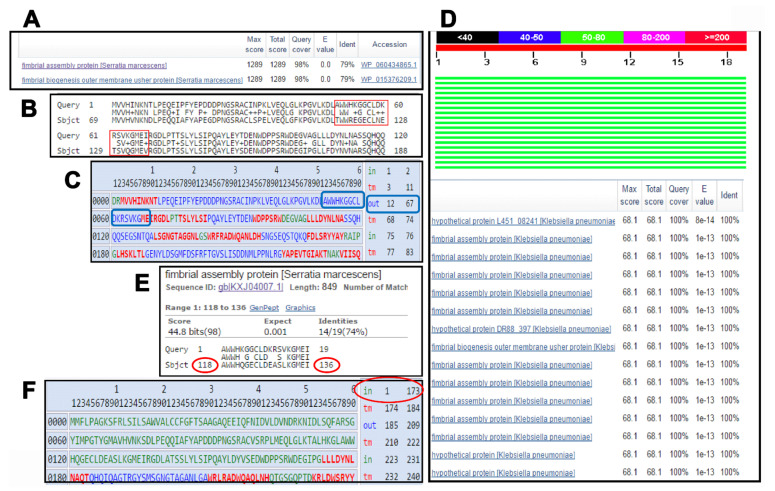
**Bioinformatic mining of the target peptide.** (**A**) result of the blast analysis of the whole *K. pneumoniae* candidate protein (KFJ72693) (gene locus tag DR88_397) when excluding the genus *Klebsiella* from the search parameters, (**B**) Alignment of the *K. pneumoniae* protein with the closest non-*Klebsiella* match from *S. marcescens*, showing the divergent region highlighted with a red box, (**C**) topology analysis of the chosen peptide region from the *K. pneumoniae* protein highlighted in blue showing its outward orientation, (**D**,**E**) blast analysis and peptide alignment using the target *K. pneumoniae* peptide as query, including then excluding *Klebsiella* from the search, respectively. The alignment shows the degree of identity with the homologous peptide from *S. marcescens* and the position of the peptide highlighted in red (**F**) topology analysis of the peptide region from *S. marcescens* highlighted in showing its inward orientation in the OM. In (**C**,**F**), the analysis was done using the PRED-TMBB server.

The proteome of *K. pneumoniae* strain ATCC 13383 was analyzed to determine the best targets for use in the ICS. Subcellular protein localization analysis using the PSORTb indicated that it had a total of 4667 proteins with 95 in the outer membrane, 2067 in the cytoplasm, 1183 in the cytoplasmic membrane, 26 extracellular, 178 periplasmic, and 1118 with unknown localization. The surface-exposed outer membrane proteins, as well as the secreted proteins are expected to be good diagnostic targets due to their high accessibility by the targeting antibodies. However, for the potential dilution effect, the detection of secreted proteins might be challenging. Thus, only the 95 outer membrane proteins were further scrutinized. No whole proteins were found to combine between the high degree of conservation among the strains of the same species and the uniqueness to the members of this species. Therefore, investigation was directed towards finding regions within the examined proteins that fulfilled the required criteria. The protein that stood out harboring promising candidate peptide regions was the hypothetical protein (KFJ72693) (gene locus tag DR88_397). The whole protein had 79% identity as *Serratia marcescens* fimbrial assembly protein (Figure 1A). Upon examining the pairwise alignment of the two proteins, it was observed that the region between aa 50 and 68 is divergent (Figure 1B). Upon checking the topology of this protein, a good portion of that region is predicted to be facing the extracellular side of the outer membrane (Figure 1C). A blast analysis using this peptide sequence against *K. pneumoniae* showed a high degree of conservation across many of the available *K. pneumoniae* strains (Figure 1D). On the other hand, upon excluding the *K. pneumoniae* from the Blast search, many hits were obtained. They included mainly *S. marcescens* (Figure 1E), which can be involved in sepsis infections. However, upon checking the topology of the homologous region in *S. marcescens*, it was predicted to be facing the intracellular side of the OM, thus not likely to react with the targeting antibodies (Figure 1F).

### 3.2. The KFJ72693 Encoding Gene (DR88_397) Is Expressed under Relevant Conditions

**Figure 2:**
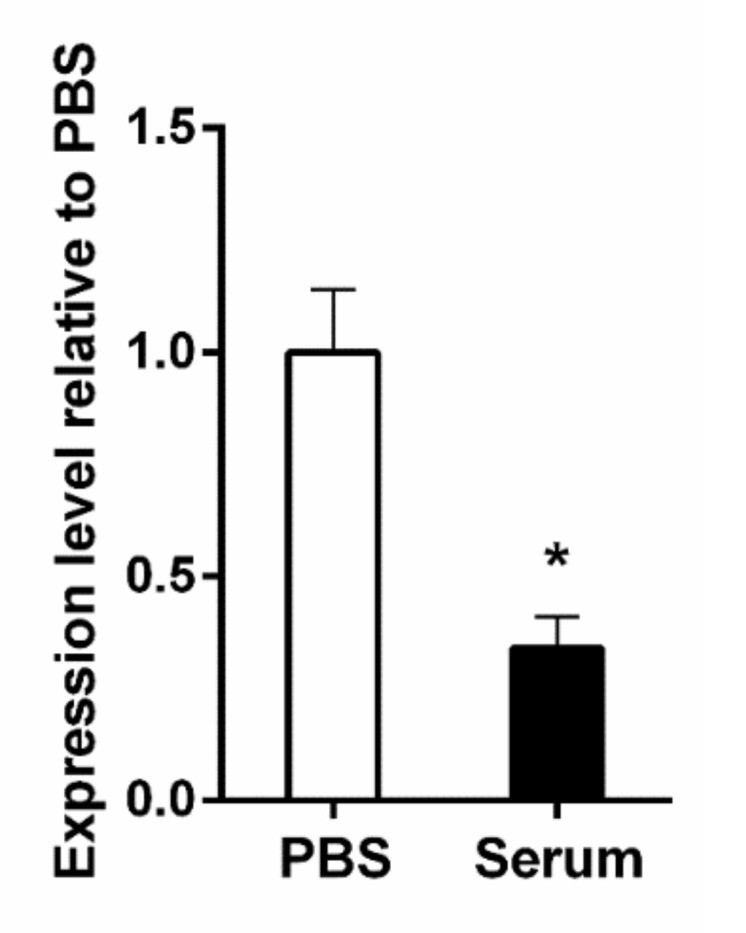
**Expression analysis of the target *K. pneumoniae* protein.** A bar graph comparing the relative expression of the target protein after incubation of the *K. pneumoniae* cells for 2 h at 37 °C in PBS versus NHS. The expression level in PBS was used as the calibrator. The data plotted represents the mean of three independent experiments, and the error bars represent the standard error. The asterisk indicates statistical significance using the student’s *t*-test (*p* ≤ 0.05).

In order to ensure that the potential target protein was indeed expressed by *K. pneumoniae*, bacterial cells grown to mid-logarithmic phase were then incubated with either PBS or NHS. Gene expression analyses of the *K. pneumoniae* gene DR88_397 indicated that it was expressed in either the presence or absence of NHS (Figure 2). Upon quantification using the ^ΔΔ^CT method with the levels of the 16S as normalizer and expression level in the presence of PBS as a calibrator, it was found that DR88_397 expression level is down regulated in the presence of serum. Therefore, the expression of the target protein was validated.

### 3.3. Evaluation of the Synthesized Peptide

Three assays were performed to assess purity, identity, and solubility. For KP_397(50-68), the purity of the peptide was assessed using HPLC and the chromatogram is presented in (Figure S1). Results in Table S1 showed a single peak at retention time (15.803) representing more than 79%. For the identity, the mass spectrum of peptide KP_397(50-68) (Figure S2) was detected at *m*/*z* [M + 2H]2 + 1101.04, [M + 3H]3 + 734.70, and [M + 4H]4 + 551.30 with a theoretical molecular weight 2201.58 Da and observed molecular weight 2201.10 Da. Upon testing the solubility of the peptide, it was found to be soluble in ultra-pure water (≤10 mg/mL) and DMSO (≤15 mg/mL).

### 3.4. The KP_397(50-68) Peptide Is Moderately Immunogenic in Mice

Using the synthetic peptides as the coating antigens, the KP_397(50-68) peptide produced a titer of 2^11^ as compared to the sham immunized mice sera. Upon using the whole cells as coating antigens, comparable titers were obtained. The purified antibodies were then pooled and concentrated to a final concentration of 430 μg/mL.

### 3.5. Successful Production of Gold Suspension in the Nanometer Range

**Figure 3:**
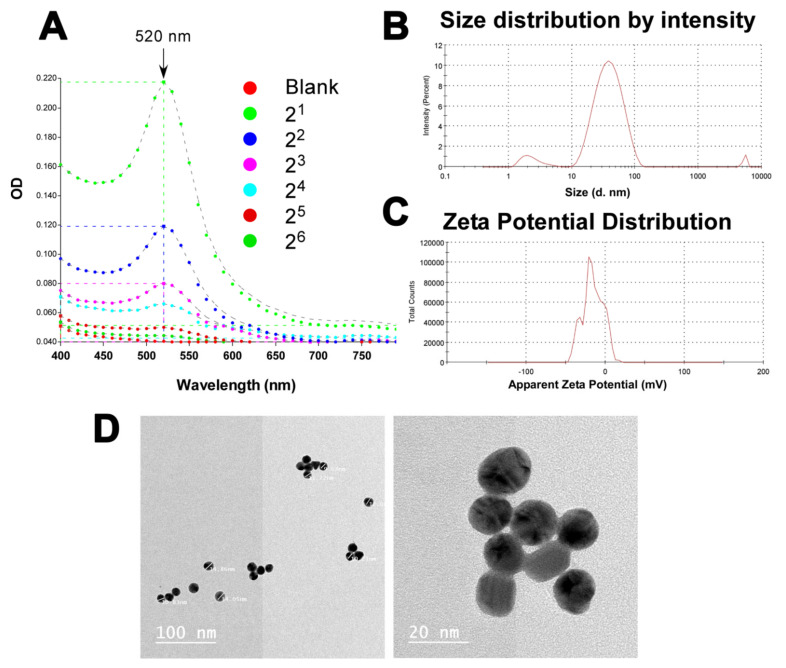
**Characterization of the synthesized GNPs.** (**A**) A schematic diagram representing the absorption spectra for two-fold serial dilutions of the GNP preparations screening for the λmax in the range between 400–800 nm. The measurements were performed in a Synergy 2 (BioTek, USA) multi-mode plate reader. (**B**,**C**) schematic diagrams showing the size (mean = 31.56 ± 1.24 nm) and the zeta potential (−32.55 ± 9.55 mV) distribution of the synthesized GNP. Measurements were done in duplicate using a Malvern Zetasizer Nano-ZS at 90° and 120° angles for size and zeta potential, respectively, at 25 °C with samples diluted 1:10 using sterile water for injection, (**D**) Representative TEM photographs of the prepared GNP at different magnifications and showing a range of sizes. The photographs were generated using a Jeol transmission electron microscope 2000 fx.

The results of the spectrophotometric scan presented in (Figure 3A) show a decrease in absorption upon dilution and maximum absorption at 520 nm, agreeing with what is normally reported for GNP suspensions [34]. The mean particle size of the nanoparticles was determined. The average particle size was 31.56 ± 1.24 nm (Figure 3B). Checking the report of the particle size distribution according to the intensity (Figure 3B), the majority (91.9%) of the particles had a diameter of 41.12 ± 20.17 nm. Another population representing 6.6% of the particles has a diameter of 2.46 ± 0.99 nm. Finally, a very small proportion (1.6%) of the particles had a diameter size of 5381 ± 322 nm. The latter proportion could represent an aggregate. This indicates that the majority of the particles in the produced suspension is in the nano meter range. The zeta potential of the GNP suspension was measured in mV by the Malvern Zetasizer Nano-ZS. The mean zeta potential value obtained was −32.55 ± 9.55 mV (Figure 3C). This indicates that the produced nano-gold suspension is stable according to the accepted range of having a zeta potential >+25 mV or <−25 mV as an indication of high degree of stability [35]. The PDI value for the analyzed GNPs was 0.409, indicating a narrow range of particle size distribution [36]. Transmission electron microscopy (TEM) was also used to give an idea about the shape of the GNPs rather than the size distribution analysis. The latter was better estimated using dynamic light scattering (DLS), as described above. Checking multiple fields of the obtained TEM images, the GNPs appeared spherical and with diameter size within the nm range (Figure 3D).

### 3.6. The Developed ICS Specifically Detects K. pneumoniae in Neonatal Sepsis Samples

**Figure 4:**
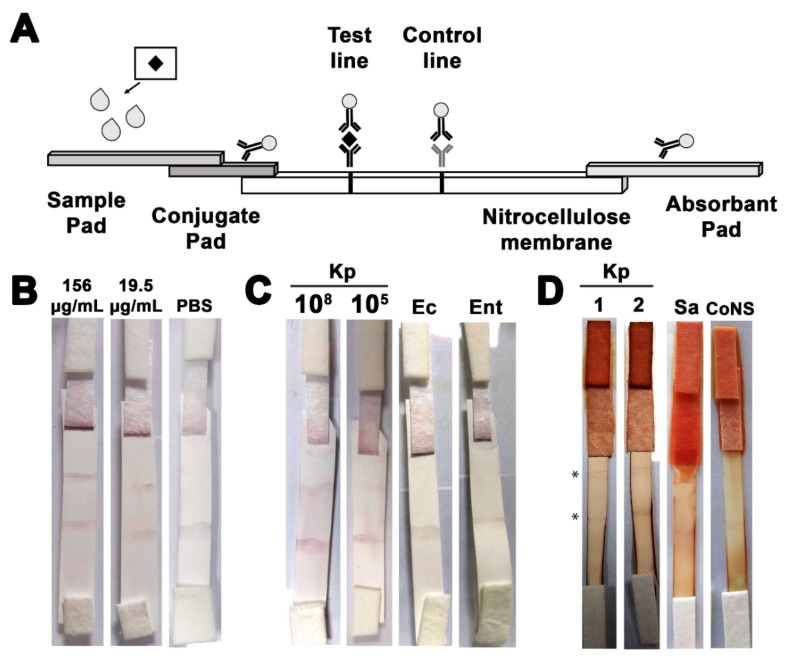
**Testing the constructed ICS.** (**A**) A schematic diagram showing the components of the ICS. The black diamond represents the target analyte in the sample, and the grey spheres represent the GNPs conjugated to the peptide-specific immunoglobulin. The grey immunoglobulin represents the Goat anti-mouse IgG control, (**B**) Representative photographs of the ICS tested with a serial dilution of the synthesized peptide. The 156 μg/mL represents the highest tested concentration, and the 19.5 μg/mL represents the limit of detection (least concentration giving a positive result). PBS was used as a negative control, (**C**) Representative photographs of the ICS tested with a serial dilution of cultured *K. pneumoniae* cells. The 108 CFU/mL represents the highest tested concentration, and the 10^5^ CFU/mL represents the limit of detection (least concentration giving a positive result). Samples of *E. coli* and *E. cloacae* (10^6^ CFU/mL) were used as negative controls, (**D**) Representative photographs of the ICS tested with a 1:10 dilution of *K. pneumoniae* neonatal sepsis blood samples. The asterisks represent the test and control lines. Sepsis blood samples positive for *S. aureus* and CoNS were used as negative controls.

The BSA stabilization assay results indicated that the least amount of protein to stabilize the synthesized GNPs was 200–300 μg/mL. This served as a reference to the amount of purified anti- KP_397(50-68) antibodies to conjugate to the GNPs. The ICS was then assembled as described in the Material and Methods section using the antibody-GNP conjugate, and diagrammatic sketch of the design of the strip is presented in (Figure 4A). The prototype ICS was first tested against a serial dilution of the purified KP_397(50-68) antigen against which the GNPs-conjugated immunoglobulins were raised. The lowest amount of the antigen that could be detected by the ICS was 19.5 µg/mL (Figure 4B). Next, the strip was challenged with suspensions of serially diluted *K. pneumoniae* cells. The strip gave a positive result with 200 µL of 10^5^ CFU/mL suspension, as shown in (Figure 4C). The development of the color on either the test or the control lines took between 15–20 min on average with both the peptides and the cells. In order to determine the specificity of the developed strips, they were challenged with bacterial species that are closely related to them and which are known to be also involved in sepsis infections. Only the *K. pneumoniae* cells produced a positive signal at the test line, while both *E. cloacae* and *E. coli* cells did not produce any positive signal confirming the specificity of the ICS (Figure 4C).

Finally, the strip was challenged with blood samples collected from neonates who were suspected of having sepsis and later gave positive blood cultures. The developed ICS gave only positive results with samples containing *K. pneumoniae*, while those infected with either *S. aureus* or coagulase-negative staphylococci (CoNS) showed negative results (Figure 4D). The average time for the development of the ICS in case of testing blood samples was 20–30 min. These results indicate that the ICS developed is fairly sensitive, specific, and could be used for the timely detection of *K. pneumoniae* in neonatal blood samples.

## 4. Discussion

Rapid and accurate diagnosis of neonatal sepsis is needed to save the lives of infants, as well as prevent antibiotic abuse and the subsequent rise of resistant strains that result from starting empirical antibiotic therapy in suspected cases [11]. ICS are ideal for this purpose due to their ease of use as point-of-care tests, their relative low cost, good sensitivity, and most importantly their rapid results, which could be obtained in minutes. A study on 2154 cases of septic shock patients showed that 83% of patients who were treated with antibiotic therapy specific to the pathogen within 30 min of the onset of symptoms survived. The mortality rate increased afterwards by 7% for each hour of delay in starting treatment [37].

ICS have been developed for the diagnosis of all sorts of infectious diseases, notably for parasitic infections [38,39,40], viral infections [41,42], and with a special focus on veterinary medicine [43,44,45]. ICS have also been developed for the detection of some bacterial pathogens, in particular foodborne pathogens and their toxins [46]. Development of ICS for the detection of human pathogens such as *Mycobacterium tuberculosis* [47], *S. aureus* [26,48], *Helicobacter pylori* [49], *H. influenzae* [50], and *Neisseria gonorrhoeae* [51] has been attempted, yet not as extensively as the previous applications.

One important factor for the success of the ICS is the selection of an ideal target that is highly expressed on the cell surface or secreted in large amounts in the extracellular environment, highly conserved in and specific to members of the target species. Due to the close relatedness between the genus *Klebsiella* and other members of the Enterobacteriaceae family of Gram-negative microorganisms, it was challenging to find a target which would achieve the above-mentioned criteria in the pool of investigated outer membrane proteins. Another challenge was finding species-specific targets that would only be detected in *K. pneumoniae* and not in other species of the genus *Klebsiella*. This problem was overcome through the choice of small synthetic peptides as targets for raising specific immunoglobulins. Although peptides offer excellent vaccine candidates in terms of tissue/target specificity, short peptides might suffer from low immunogenicity and the inability to stimulate the necessary components of immune response [52]. Also, when peptides are taken out of the context of a whole protein as a delivery system, they might not elicit the same immune response due to conformational changes [52]. That is why peptides were assessed from the proteins achieving the highest numbers/scores of immunogenic epitopes based on the analysis through the Immune Epitope Database. We also confirmed that the choice was finally limited to those peptides which were topographically exposed in the outer membrane protein itself to avoid inaccessible epitopes buried in transmembrane domains. Despite all of these efforts to choose an ideal peptide target, immunoglobulin titers obtained were still very moderate. The chosen peptide target is part of a hypothetical protein of unknown function in *K. pneumoniae*, however, blast search indicated that the protein is homologous to a fimbrial assembly protein in *S. marcescens*. Expression analysis indicated that the expression of the target protein was downregulated in presence of serum, a condition tested to mimic what would happen upon the introduction of *K. pneumoniae* cells to the bloodstream in case of sepsis. Fimbrial expression-regulating genes studied before showed a great fluctuation in their expression levels based upon the host environment of *K. pneumoniae* cells, and were downregulated in many cases [53]. In all cases, the experiment served its purpose by showing that the hypothetical *K. pneumoniae* was actively expressed.

Polyclonal antibodies raised through the vaccination of mice was chosen for the development of the ICS. Polyclonal antibodies have the advantage of short production time, inexpensive to make, high affinity, and less sensitivity to antigen changes than monoclonal antibodies. However, different batches of the same polyclonal antibody could be highly variable, and its reactivity with multiple epitopes could affect the specificity of the ICS [54]. For the purpose of this work, polyclonal antibodies served as a good proof-of-principle and further optimization could be done using the more highly specific monoclonal antibodies in future work.

Immunoglobulin-GNP conjugates were successfully produced to be used as the indicator system for the ICS. The use of GNP in lateral flow immunoassays (LFIAs) is well established and amounts to more than 75% of the application as a label [46]. Although there are currently many alternatives which could offer higher sensitivity, GNPs are widely used because they are economical, easy to produce, and are intensely colored [55]. The performance and sensitivity of LFIAs greatly depends of the size and shape of the used GNPs. The average size of the GNPs described in this work was ~ 31 nm and they had the shape of nanospheres. Larger GNPs are usually less stable and require antibodies with high concentration for their stabilization and to produce conjugates [56]. Shapes other than spheres, such as nanorods, nanostars, and nanopopcorns offer higher stability due to their 3-dimensional complex structures; however, spherical NPs provide a larger surface area for the immobilization of antibodies [57]. The GNPs used in the current study exhibited a zeta potential of −32.55 ± 9.55 mV, which indicative a good stability since it is within the acceptable range of > +25 mV or < −25 mV [35] and similar to the previously described values for nano-particles [58,59]. However, it is worth mentioning that the zeta potential in the current study was determined in a solution that was diluted 1:10; this dilution could have an effect on the value of the obtained zeta potential [60,61]. Having values within the acceptable range for stability despite dilution was a good indication of the stability of the obtained solution.

In this work, the developed strip was able to specifically detect *K. pneumoniae* in neonatal septic blood samples with a sensitivity of 10^5^ CFU/mL unprocessed blood samples, apart from dilution, in an average of 15–20 min. Although the visibility of the positive test line was reduced a little due to the red color of blood, the experiment was intended to test the possibility of testing the blood samples in economically challenged settings with limited resources to elaborately process the samples prior to testing. It is expected that an addition of a simple blood decolorization step could greatly enhance the readability of the test result without affecting the sensitivity of the ICS nor the integrity of the target cells [62].

Some previous trials for the detection of *Klebsiella* species through LFIAs are documented in the literature, notably in food samples [63,64]. In these reports, processing of food samples followed by an incubation of 24 h was required. In addition, the ICS constructed also gave a positive result with other genera particularly with *Serratia*, as well as with *Enterobacter, Citrobacter,* and *E. coli*. This required further confirmation of the genus using urease test in some instances. As for the sensitivity, reliable results were obtained with ≥ 10^6^ CFU. Another group investigated the use of an ICS for the rapid detection of capsular serotype type of *K. pneumoniae* in pus samples from liver abscess and positive blood cultures from sepsis cases [65]. The sample was obtained from blood culture bottles, after incubation, then using a subsequently isolated single colony from the culture media plates.

## 5. Conclusions

To the knowledge of the authors of this work, this is the first report of an ICS for the direct testing of septicemia blood samples without any prior culture or sample processing. The prototype ICS showed promising results in terms of the test to interpretation time, specificity, and sensitivity. Further optimization would present this kit as an excellent point-of-care test that could significantly contribute to the saving of infant lives in local as well as international settings.

## Data Availability

All the data regarding the study is available through this manuscript. No additional reporting is available elsewhere.

## Supplementary Materials

The following are available online at https://www.mdpi.com/article/10.3390/pharmaceutics13081141/s1, Figure S1: HPLC chromatogram for KP_397(50-68), Figure S2: Mass spectrum of peptide KP_397(50-68), Table S1: Analysis of the peaks detected in the HPLC of KP_397(50-68).
