# Supplementary material for: Development of an Immunochromatographic Strip Using Conjugated Gold Nanoparticles for the Rapid Detection of Klebsiella pneumoniae Causing Neonatal Sepsis
Source: Pharmaceutics. 2021 Jul 26;13(8):1141. doi: 10.3390/pharmaceutics13081141 (PMC8401635; doi:10.3390/pharmaceutics13081141)
Supplement: Supplementary file 1 [file pharmaceutics-13-01141-s001.zip › pharmaceutics-1261021-supplementary.pdf]

pharmaceutics

Supplementary Materials: Development of an Immunochromatographic Strip Using Conjugated Gold Nanoparticles for the Rapid Detection of *Klebsiella pneumoniae* Causing Neonatal Sepsis

Noha M. Elhosseiny, Tamer M. Samir, Aliaa A. Ali, Amani A. El-Kholy and Ahmed S. Attia

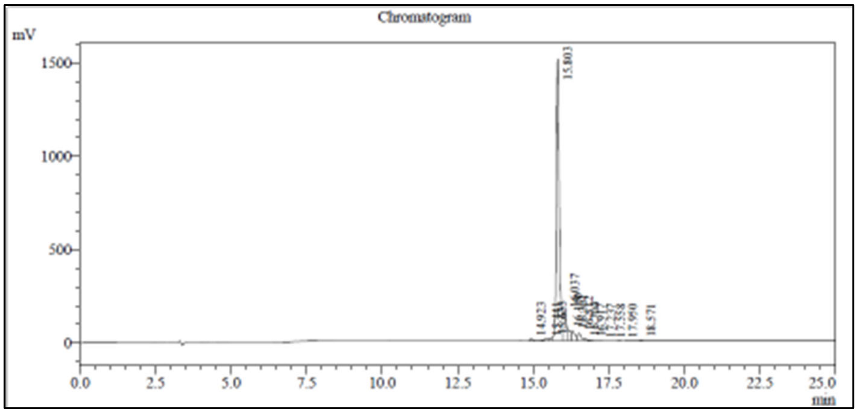

Figure S1. HPLC chromatogram showing one major peak for KP_397(50-68) at retention time of 15.8 min. Analysis was carried out using AlltimaTM C18 5 μm, 4.6 250 mm column, mobile phase A: 0.065% TFA in water & B:0.05% TFA in acetonitrile, at flow rate of 1 ml/min and detection at 220 nm.

Table S1. Analysis of the peaks detected in the HPLC of KP_397(50-68).

Peak#	Ret. Time	Area	Height	Area %
1	14.923	73772	9004	0.568
2	15.441	118940	9985	0.916
3	15.633	101532	18130	0.782
4	15.803	10299956	1508188	79.296
5	16.037	1126114	162584	8.670
6	16.150	339909	57774	2.617
7	16.301	429368	51494	3.306
8	16.512	343949	39608	2.648
9	16.704	78667	9778	0.606
10	16.917	35591	3302	0.274
11	17.237	17034	1568	0.131
12	17.558	5119	436	0.039
13	17.950	136	34	0.001
14	18.571	19167	3321	0.148
Total		12989254	1875204	100.000

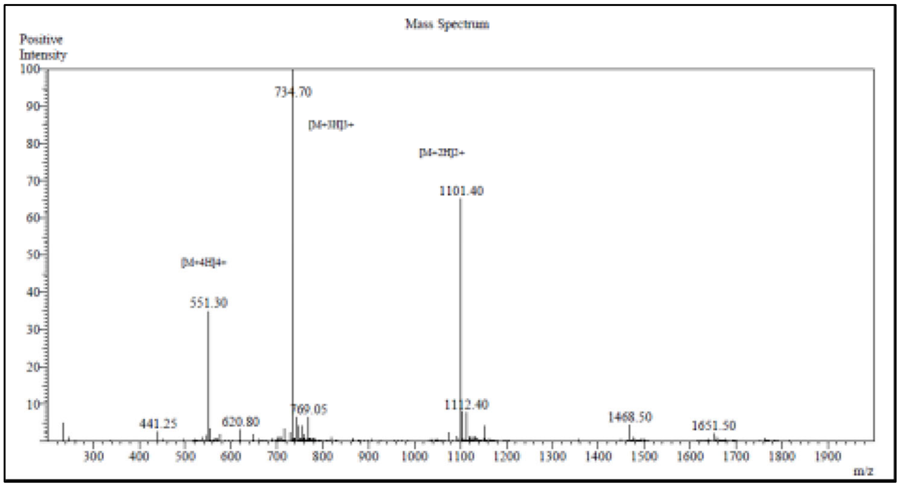

Figure S2. Mass spectrum of peptide KP_397(50-68) detected at m/z $[M+2H]^{2+}$ 1101.04, $[M+3H]^{3+}$ 734.70, and $[M+4H]^{4+}$ 551.30 theoretical molecular weight 2201.58 Da and observed molecular weight 2201.10 Da). Sample was dissolved in 50% methanol (*v*/*v*), analysis was carried out by direct infusion using electrospray ionization in positive mode (Nebulizing Gas Flow: 1.5L/min, Drying Gas Flow :5 L/min, T. Flow :0.2 ml/min, B. conc :50%H2O/50%MeOH, CDL Temp: 250°C, and Block Temp:200°C.
